# Uninterrupted Dabigatran Administration Provides Greater Inhibition against Intracardiac Activation of Hemostasis as Compared to Vitamin K Antagonists during Cryoballoon Catheter Ablation of Atrial Fibrillation

**DOI:** 10.3390/jcm9093050

**Published:** 2020-09-22

**Authors:** Zsuzsa Bagoly, Orsolya Hajas, Réka Urbancsek, Alexandra Kiss, Edit Fiak, Ferenc Sarkady, Noémi Klára Tóth, Rita Orbán-Kálmándi, Kitti Bernadett Kovács, László Nagy, Attila Nagy, János Kappelmayer, László Csiba, Zoltán Csanádi

**Affiliations:** 1 Division of Clinical Laboratory Sciences, Department of Laboratory Medicine, Faculty of Medicine, University of Debrecen, 4032 Debrecen, Hungary; bagoly@med.unideb.hu (Z.B.); sarkadyf@gmail.com (F.S.); tnk@med.unideb.hu (N.K.T.); kalmandi.rita@med.unideb.hu (R.O.-K.); 2 Department of Cardiology, Faculty of Medicine, University of Debrecen, 4032 Debrecen, Hungary; orsolya.hajas@gmail.com (O.H.); urbancsekreka@gmail.com (R.U.); kiss.alexandra@hotmail.com (A.K.); editfiak12@gmail.com (E.F.); nagylaszlo69@gmail.com (L.N.); 3 MTA-DE Cerebrovascular and Neurodegenerative Research Group, 4032 Debrecen, Hungary; csiba@med.unideb.hu; 4 Department of Neurology, Faculty of Medicine, University of Debrecen, 4032 Debrecen, Hungary; kovacskitti985@gmail.com; 5 Department of Preventive Medicine, Faculty of Public Health, University of Debrecen, 4028 Debrecen, Hungary; nagy.attila@sph.unideb.hu; 6 Department of Laboratory Medicine, Faculty of Medicine, University of Debrecen, 4032 Debrecen, Hungary; kappelmayer@med.unideb.hu

**Keywords:** atrial fibrillation, cryoballoon ablation, dabigatran, vitamin K antagonists

## Abstract

Background. Cerebral thromboembolism is a rare but feared complication of transcatheter ablation in patients with atrial fibrillation (AF). Here, we aimed to test which pre-procedural anticoagulation strategy results in less intracardiac activation of hemostasis during ablation. Patients and methods. In this observational study, 54 paroxysmal/persistent AF patients undergoing cryoballoon ablation were grouped according to their periprocedural anticoagulation strategy: no anticoagulation (oral anticoagulation (OAC) free; *n* = 24), uninterrupted vitamin K antagonists (VKA) (*n* = 11), uninterrupted dabigatran (*n* = 17). Blood was drawn from the left atrium before and immediately after the ablation procedure. Cryoablations were performed according to standard protocols, during which heparin was administered. Heparin-insensitive markers of hemostasis and endothelial damage were tested from intracardiac samples: D-dimer, quantitative fibrin monomer (FM), plasmin-antiplasmin complex (PAP), von Willebrand factor (VWF) antigen, chromogenic factor VIII (FVIII) activity. Results. D-dimer increased significantly in all groups post-ablation, with lowest levels in the dabigatran group (median [interquartile range]: 0.27 [0.36] vs. 1.09 [1.30] and 0.74 [0.26] mg/L in OAC free and uninterrupted VKA groups, respectively, *p* < 0.001). PAP levels were parallel to this observation. Post-ablation FM levels were elevated in OAC free (26.34 [30.04] mg/L) and VKA groups (10.12 [16.01] mg/L), but remained below cut-off in all patients on dabigatran (3.98 [2.0] mg/L; *p* < 0.001). VWF antigen and FVIII activity increased similarly post-ablation in all groups, suggesting comparable procedure-related endothelial damage. Conclusion. Dabigatran provides greater inhibition against intracardiac activation of hemostasis as compared to VKAs during cryoballoon catheter ablation.

## 1. Introduction

Transcatheter isolation of pulmonary veins (PVI) is an established therapy for atrial fibrillation (AF) [1]. Although point-by-point radiofrequency ablation is the traditional method of PVI, similar success rates, but shorter procedure times, have been demonstrated with cryoballoon ablation (CBA) in multiple trials [2,3,4], which has, therefore, become the preferred technique of PVI in many centers. Regardless of the ablation methodology, periprocedural antithrombotic treatment is crucial in preventing procedure-related thromboembolism, a potentially life-threatening complication of left atrial (LA) ablation [5,6,7,8].

While the use of non-fractionated heparin to reach a target activated clotting time (ACT) of 300 s or above during catheter dwelling in LA is a standard practice, optimal oral anticoagulation (OAC) before the ablation has been a matter of long-term debate. The risk of a potentially life-threatening bleeding complication inherent to transseptal catheterization, catheter manipulation, and the delivery of ablative energy in the thin-wall LA argue against uninterrupted administration of any OAC in the absence of a specific antidote capable of restoring hemostasis in case of a bleeding complication. Despite these concerns, observational and randomized clinical trials have demonstrated the safety or even superiority of uninterrupted administration of vitamin K antagonists (VKA) [9,10,11]. Recently, different Non-Vitamin-K Oral Anticoagulants (NOACs) have also been studied in this clinical scenario. Several trials of various size and designs reported similar efficacy and comparable or improved safety outcomes with different factor Xa (FXa) inhibitors as compared with VKAs for periprocedural use [12,13,14,15]. However, conflicting results have been published with the thrombin inhibitor dabigatran. A multicenter non-randomized observational study found significantly increased rates of both thromboembolic and bleeding complications with interrupted administration of dabigatran as compared with non-interrupted VKA [16]. On the contrary, uninterrupted administration of dabigatran in randomized trials [17,18] was associated with significantly less bleeding complications as compared with continuous VKA administration. Recent studies suggest that in AF patients, intracardiac blood sampling revealing local conditions of hemostasis activation and endothelial damage might reflect the thrombogenic state more precisely, as blood sampling from the systemic circulation might not be able to show subtle, but significant local changes [19,20,21,22]. However, potential differences between various OACs to inhibit hemostasis activation in the settings of left atrial ablation, most probably due to the difficulty of intracardiac sampling, have not been directly tested from intracardiac blood samples as of yet.

Herein, we studied the effect of different pre-procedural antithrombotic treatment strategies on hemostasis and fibrinolysis as assessed in LA blood samples before and after the ablation in patients undergoing CBA for paroxysmal/persistent AF. Three different peri-procedural antithrombotic treatment schemes were compared: 1, no pre-ablation OAC administration; 2, uninterrupted VKA administration, and 3, uninterrupted dabigatran administration.

## 2. Experimental Section

### 2.1. Study Population

In this single-centered observational study, consecutive patients, who all underwent radiofrequency ablation due to symptomatic paroxysmal or persistent AF, were included based on the following criteria: age 18–75 years, willingness to sign an informed consent, documented, symptomatic paroxysmal, or persistent AF, and failure of at least one antiarrhythmic drug. Exclusion criteria were: long-standing persistent AF, valvular heart disease, reversible cause of AF (e.g., hyperthyroidism), previous cardiac surgery, presence of a left atrial thrombus, left ventricular ejection fraction (LVEF) ≤30%, heart failure of New York Heart Association functional classification (NYHA) class III or IV, history of transient ischemic attack (TIA) or ischemic stroke, documented carotid stenosis, myocardial infarction or unstable angina within the past 3 months, severe chronic obstructive pulmonary disease, known bleeding diathesis or thrombotic disorder, acute inflammatory disease, contraindication to oral anticoagulation, and pregnancy.

The study design and its execution were in accordance with the ethical principles of the Declaration of Helsinki. The study was approved by the Regional and Institutional Ethics Committee of the University of Debrecen and the National Medical Research Council Ethics Committee (ETT TUKEB 9702-2/2016/EKU). All patients signed an informed consent form prior to inclusion.

### 2.2. Pre-Procedure Anticoagulation and Ablation Procedure

**Figure 1:**
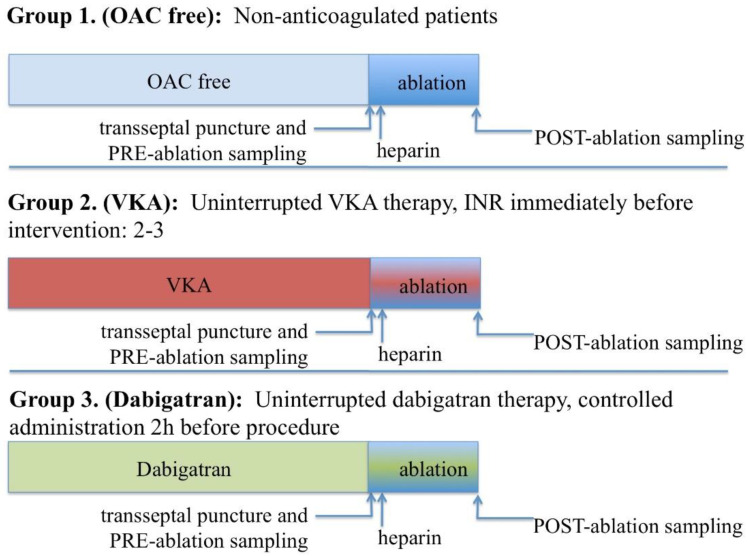
Study groups according to pre-procedural anticoagulation strategy. INR: International Normalised Ratio, OAC: oral anticoagulant, VKA: vitamin K antagonist.

All medications with a potential inhibitory effect on platelet activity were discontinued before the procedure for a period of at least 7 days. Transesophageal echocardiography was performed within 24 h prior to the procedure to rule out the possibility of a cardiac thrombus. Pre-procedural anticoagulation was kept unchanged as compared to the treatment patients had been receiving at the time the ablation was scheduled, and thereby included one of three schemes (Figure 1):

1. No anticoagulation (OAC free group). All patients on no anticoagulation were considered low risk for thromboembolism (CHA_2_DS_2_-VASC-score 0–2).
2. Uninterrupted vitamin K antagonist for at least 30 days pre-ablation to maintain a therapeutic international normalised ratio (INR) between 2–3, which was confirmed on the morning of the procedure (VKA group). In case the morning INR level was measured out of the therapeutic range, ablation was postponed and VKA dose adjusted as required.
3. Dabigatran 150 mg BID for at least 30 days with the last dose given 2 h prior to the procedure (Dabigatran group). Dabigatran was administered to all patients exactly 2 h before the scheduled start of the ablation, by the form of controlled pill intake as inspected by a nurse.

Procedures were performed under conscious sedation, using midazolam and fentanyl according to standard practice at our institution [22]. Three punctures of the right femoral vein were performed using the Seldinger technique and introducers with side arms were placed in the vein. A multipolar electrode catheter was placed in the coronary sinus and an intracardiac echocardiography (ICE) catheter in the right atrium to guide transseptal puncture and positioning of the CB catheter. A single transseptal puncture was performed with a Brockenborough needle and a Mullins sheath was advanced in the LA. Immediately after transseptal puncture and pre-ablation blood sample collection, a 150 IU/kg body weight intravenous heparin bolus was given, followed by a continuous infusion to maintain a minimum target activated clotting time level of >300 s. LA sheaths were flushed continuously with heparinized saline at a steady rate of around 30 mL/h, and in case of hypotonia, additional fluid replacement was allowed throughout the procedure.

All PVI procedures were carried out with the 28 mm second-generation CB (Arctic Front Advance; Medtronic Inc., Minneapolis, MN, USA). The Mullins sheath was replaced for the deflectable 12-Fr long FlexCath sheath and introduced into the LA over a stiff long guidewire. An 8-pole, circular electrode catheter was advanced through the lumen of the cryoballoon and used as a guidewire to cannulate specific side branches of the PVs to allow the continuous monitoring of PV electrograms during each freezing cycle. The balloon was manipulated to obtain the potentially most antral position while maintaining a sufficient seal of the vein as assessed by contrast injection and ICE Doppler. Two freezing cycles of 3–4 min in duration were usually applied in each PV based on the achieved temperature and the time to PVI. Temperatures between −40 °C and −55 °C were considered suitable for the procedure. Cryoapplication was terminated in case of lower values in order to minimize the risk of collateral damage. Procedural end-point was PVI, defined as PV-LA entrance block verified with pacing maneuvers according to standard practice. Whenever necessary, sinus rhythm was restored by cardioversion at the end of the ablation procedure.

### 2.3. Blood Sampling and Laboratory Investigations

Preablation blood samples were collected through the Mullins sheath from the LA immediately after transseptal puncture and the removal of the dilator, before the intravenous administration of unfractionated heparin [21,22]. Post-ablation blood samples were drawn through the LA sheath after removal of the CB catheter, immediately after the last ablation was completed. Forty-five ml blood samples were collected, of which the first 15 mL was discarded to exclude intra-sheath activation of hemostasis. Blood was drawn into vacutainer tubes (tubes containing 0.109 M sodium citrate, and tubes containing no anticoagulant with polymer gel separator: SST tubes, Becton Dickinson, Franklin Lakes, NJ, USA). Citrated blood samples were centrifuged twice at 1500 *g*, room temperature for 15 min, while SST tubes were centrifuged once at 2000× *g*, at room temperature, for 20 min. Plasma and serum samples were aliquoted and stored at −80 °C until further analysis. Screening tests of hemostasis (prothrombin time, activated partial thromboplastin time, thrombin time) and fibrinogen levels (based on Clauss method) were assessed from freshly separated plasma samples using standard methods (Siemens Healthcare Diagnostic Products, Marburg, Germany). Stored plasma samples were used to perform specific hemostasis and fibrinolysis assays, all measurements were carried out by investigators blinded to clinical data. Quantitative D-dimer levels were measured by a particle-enhanced, immuno-turbidimetric assay (Innovance D-dimer) on a BCS coagulometer following the manufacturer’s instructions (Siemens Healthcare Diagnostic Products, Marburg, Germany). The levels of von Willebrand factor (VWF) antigen, chromogenic factor VIII (FVIII) activity, and α_2_-plasmin inhibitor (α_2_-PI) activity were measured by commercially available methods on a BCS coagulometer (Siemens Healthcare Diagnostic Products, Marburg, Germany). The levels of plasmin-α2-antiplasmin (PAP) complex were measured using an ELISA test (Technozym PAP complex ELISA kit, Technoclone, Vienna, Austria). Soluble fibrin monomer (FM) levels were determined using the Liatest FM assay (Diagnostica Stago, Asnieres, France). High sensitivity C-reactive protein (CRP) was measured from stored serum samples by routine methods (Roche Diagnostics, Mannheim, Germany). A direct thrombin inhibitor assay was used to assess dabigatran peak levels, as measured from antecubital vein blood samples drawn at the beginning of the ablation procedure (INNOVANCE DTI, Siemens Healthcare Diagnostic Products, Marburg, Germany; reference range in local laboratory: 64–443 μg/mL) [23].

### 2.4. Statistical Analysis

Statistical analysis was carried out using GraphPad Prism Software version 6.0 (La Jolla, CA, USA) and the Statistical Package for Social Sciences (SPSS, Release 26.0, Chicago, IL, USA). Normality of the data was evaluated by the Shapiro-Wilk and the D’Agostino and Pearson omnibus tests. A paired t-test or Wilcoxon matched-pairs rank-sum test was performed when comparing results obtained from pre-ablation and post-ablation intracardiac samples. ANOVA with Bonferroni post-hoc test or Kruskal-Wallis test using Dunn’s-Bonferroni post-hoc test was applied for multiple comparisons of unpaired data. In order to adjust for covariates associated with selection, analysis of covariance (ANCOVA) was performed (after logarithmic transformation of data when necessary). Differences between categorical variables were studied using the χ^2^ or Fisher’s exact test. *p* < 0.05 was considered statistically significant.

## 3. Results

### 3.1. Baseline Patient and Procedure Characteristics

A total of 52 patients were enrolled in the study. No pre-ablation anticoagulation was applied in 24, uninterrupted VKA in 11, and uninterrupted dabigatran in 17 patients. No difference in baseline patient characteristics or in LA dwelling times were found (Table 1). No thromboembolic or major bleeding complications occurred in any of the patient groups. No patients experienced cerebral events during or after the procedure.

### 3.2. Intracardiac Activation of Hemostasis and Fibrinolysis in AF Patients before and after the Ablation Procedure: Effect of Different Pre-Procedural Anticoagulation Strategies

**Figure 2:**
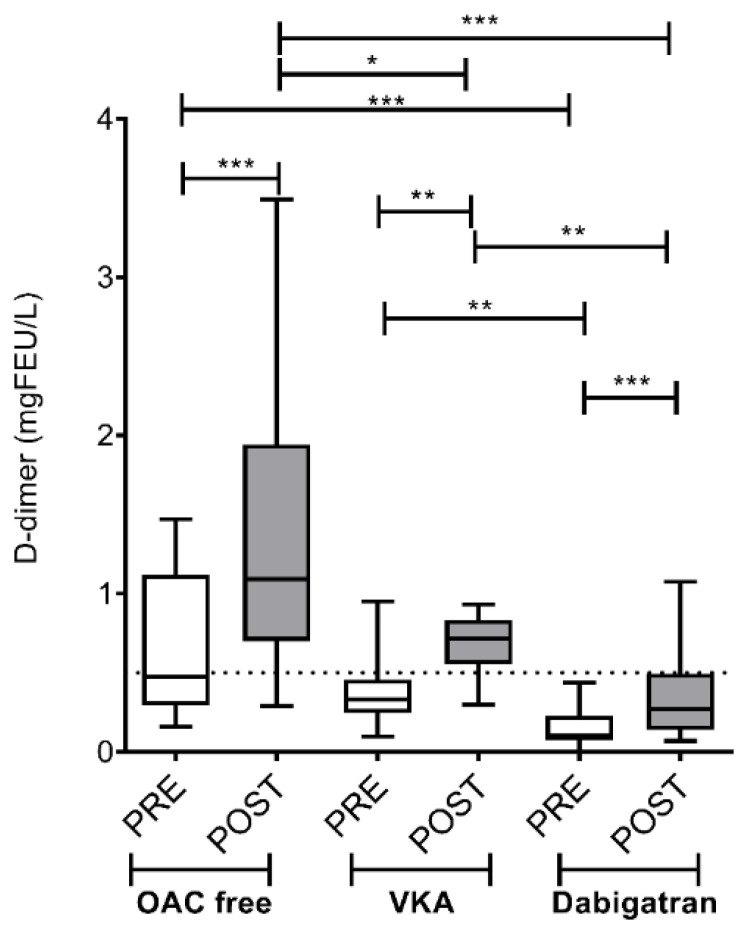
Left atrium D-dimer levels before and after cryoballoon ablation procedure in AF patients on various pre-procedural anticoagulation strategies. Box and whisker plots indicate median, interquartile range, and total range. Dashed lines indicate diagnostic cut-off level for venous thromboembolic events (0.5 mg/L). PRE (empty bars): pre-ablation, POST (solid bars): post-ablation, OAC: oral anticoagulant, VKA: vitamin K antagonist. * *p* < 0.05, ** *p* < 0.01, *** *p* < 0.001. Statistical significance remained essentially the same when assessed after controlling for the effect of covariates associated with selection (age, sex) between treatment groups.

D-dimer levels increased significantly in LA blood samples in all three groups after ablation (Figure 2). Measured values (median; IQR = interquartile range) before vs. after ablation were (0.48; IQR 0.81 vs. 1.09; IQR 1.30 mg/L, *p* < 0.001) in the OAC free group, (0.33; IQR 0.21 vs. 0.74; IQR 0.26 mg/L, *p* < 0.01) in the VKA group and (0.10; IQR 0.20 vs. 0.27; IQR 0.36 mg/L, *p* < 0.001) in the dabigatran group. Both pre- and post-ablation values were significantly lower in patients on dabigatran as compared to what was obtained in the OAC free and in the VKA groups (*p* < 0.01). Of note, post-ablation levels of D-dimer did not exceed the 0.5 mg/L cut-off in patients treated with dabigatran.

**Figure 3:**
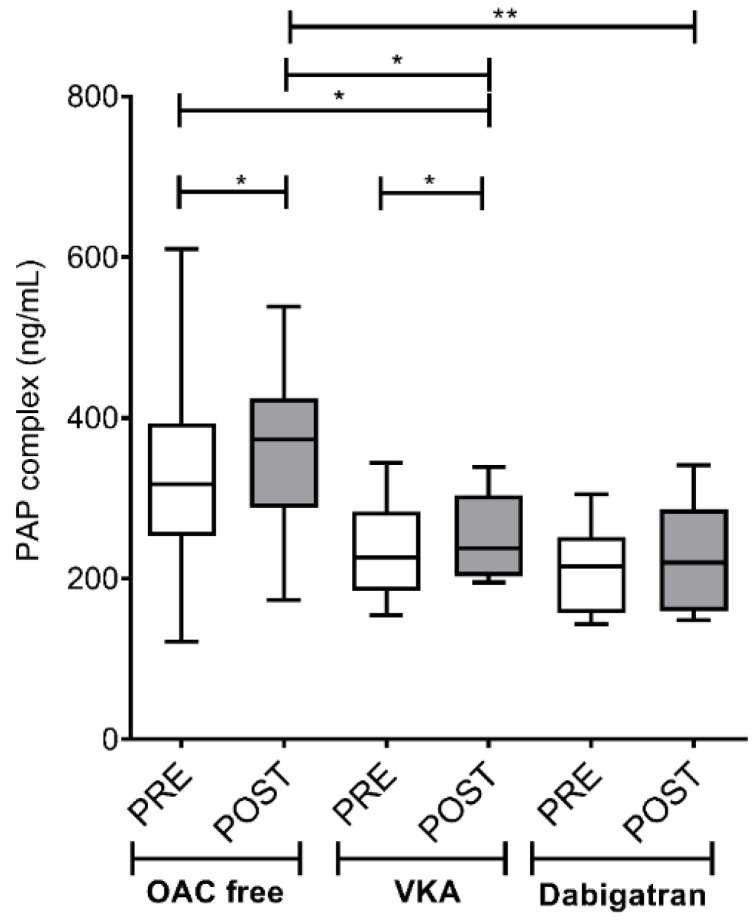
Left atrium plasmin-antiplasmin (PAP)-complex levels before and after cryoballoon ablation procedure in AF patients on various pre-procedural anticoagulation strategies. Box and whisker plots indicate median, interquartile range, and total range. PRE (empty bars): pre-ablation, POST (solid bars): post-ablation, OAC: oral anticoagulant, VKA: vitamin K antagonist. * *p* < 0.05, ** *p* < 0.01. Statistical significance remained essentially the same when assessed after controlling for the effect of covariates associated with selection (age, sex) between treatment groups.

Similarly to D-dimer, PAP complex levels increased significantly after the ablation in both the OAC free (317.57; IQR 140.43 vs. 373.07; IQR 144.93 ng/mL, *p* < 0.05) and the VKA groups (237.56; IQR 102.77 vs. 253.88; IQR 99.51 ng/mL, *p* < 0.05), but only a non-significant trend was observed in the dabigatran group (216.82; IQR 104.33 vs. 235.67; IQR 142.89 ng/mL, *p* > 0.05) (Figure 3). Of note, significantly increased post-ablation values were found in patients not treated with any OAC as compared to those on VKA (*p* < 0.05) or on dabigatran (*p* < 0.01).

**Figure 4:**
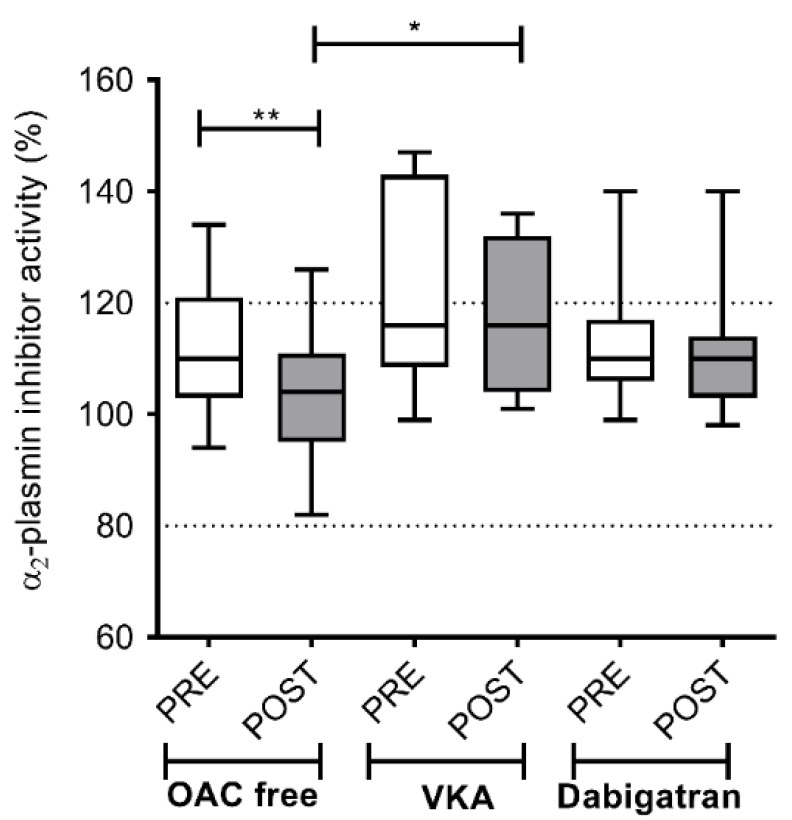
Left atrium α_2_- plasmin inhibitor activity levels before and after cryoballoon ablation procedure in AF patients on various pre-procedural anticoagulation strategies. Box and whisker plots indicate median, interquartile range, and total range. Dashed lines indicate lower and upper limit of reference interval (80–120%). PRE (empty bars): pre-ablation, POST (solid bars): post-ablation, OAC: oral anticoagulant, VKA: vitamin K antagonist. * *p* < 0.05, ** *p* < 0.01. Statistical significance remained essentially the same when assessed after controlling for the effect of covariates associated with selection (age, sex) between treatment groups.

**Figure 5:**
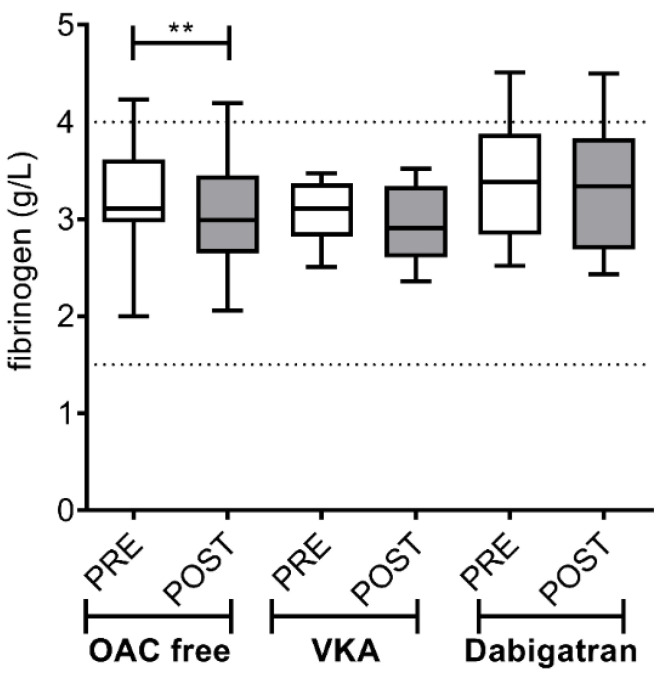
Left atrium fibrinogen levels before and after cryoballoon ablation procedure in AF patients on various pre-procedural anticoagulation strategies. Box and whisker plots indicate median, interquartile range, and total range. Dashed lines indicate lower and upper limit of reference interval (1.5–4 g/L). PRE (empty bars): pre-ablation, POST (solid bars): post-ablation, OAC: oral anticoagulant, VKA: vitamin K antagonist. ** *p* < 0.01.

A significant decrease in α2-PI activity, indicating enhanced fibrinolysis post-ablation, was demonstrated in the OAC free group (110; IQR 18 vs. 104; IQR 16%; *p* < 0.01) but not in patients on either VKA or dabigatran therapy (Figure 4). In line with these results, a significant consumption of fibrinogen after the ablation was only observed in the OAC free group (3.11; IQR 0.65 vs. 2.99; IQR 0.81 g/L; *p* < 0.01), but not in patients treated with any pre-procedural OAC (Figure 5).

**Figure 6:**
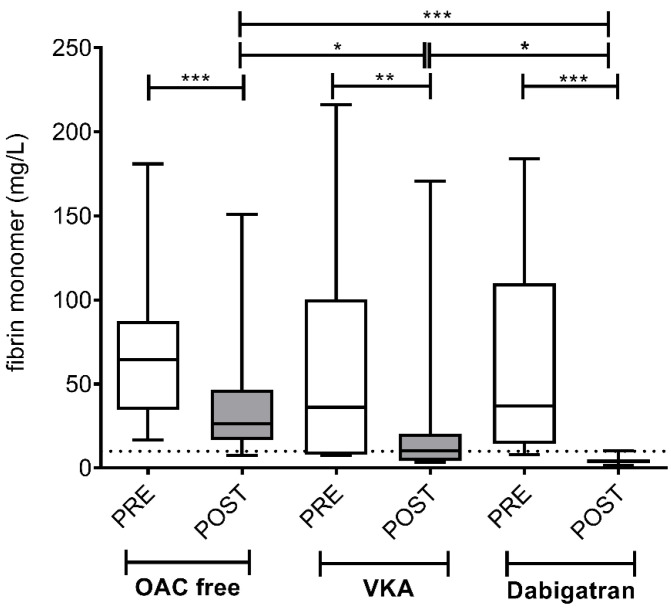
Left atrium fibrin monomer levels before and after cryoballoon ablation procedure in AF patients on various pre-procedural anticoagulation strategies. Box and whisker plots indicate median, interquartile range, and total range. Dashed lines indicate diagnostic cut-off level for prethrombotic conditions (10 mg/L). PRE (empty bars): pre-ablation, POST (solid bars): post-ablation, OAC: oral anticoagulant, VKA: vitamin K antagonist. * *p* < 0.05, ** *p* < 0.01, *** *p* < 0.001. Statistical significance remained essentially the same when assessed after controlling for the effect of covariates associated with selection (age, sex) between treatment groups.

Similarly to earlier published studies, pre-ablation median and IQR levels of fibrin monomers were above the upper limit of reference in all groups, due to hemostasis activation related to the catheterization procedure and left atrial sampling [21] (Figure 6). Due to the short (t(1/2) =2.3 h) half-life of this protein [24], a significant decrease was observed after the ablation procedure in all patient groups (OAC free group: 64.35; IQR 52.83 vs. 26.34; IQR 30.04 mg/L; *p* < 0.001; VKA group: 36.14; IQR 92.56 vs. 10.12; IQR 16.01 mg/L; *p* < 0.01; dabigatran group: 38.37; IQR 153.06 vs. 3.98; IQR 2.0 mg/L; *p* < 0.001). Of note, in case of the dabigatran group, remarkably low levels of fibrin monomers were found post-ablation. In all patients of the dabigatran group, fibrin monomer levels returned to below the limit of reference of coagulation activation, indicating significantly lower additional hemostasis activation during the ablation procedure in this patient group as compared to the VKA group and the OAC free group. In the OAC free group, post-ablation fibrin monomer levels were mostly above the reference range, suggesting a lasting activation of coagulation during the ablation procedure.

### 3.3. Local Endothelial Activation in the Left Atrium of AF Patients before and after the Ablation Procedure

**Figure 7:**
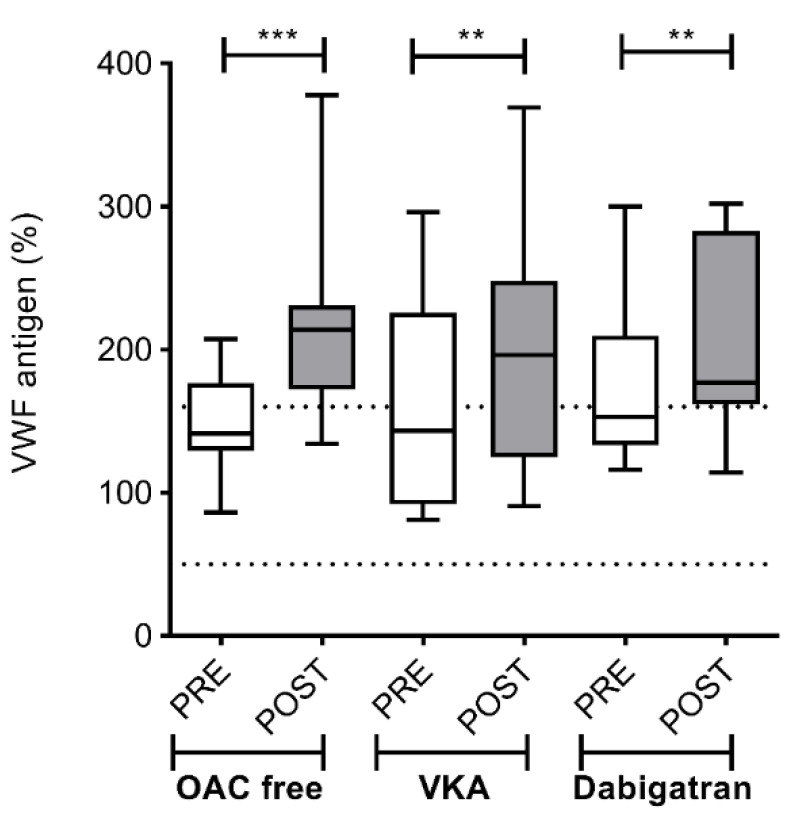
Left atrium von Willebrand factor (VWF) antigen levels before and after cryoballoon ablation procedure in AF patients on various pre-procedural anticoagulation strategies. Box and whisker plots indicate median, interquartile range, and total range. Dashed lines indicate lower and upper limit of reference interval (50–160%). PRE (empty bars): pre-ablation, POST (solid bars): post-ablation, OAC: oral anticoagulant, VKA: vitamin K antagonist. ** *p* < 0.01, *** *p* < 0.001.

VWF antigen levels showed a similar increase in all groups post-ablation as compared to pre-ablation levels, suggesting comparable procedure-related endothelial damage (OAC free: 138.6; IQR 38.83 vs. 214.1; IQR 58.55%; *p* < 0.001; VKA: 138.9; IQR 133.8 vs. 196.1; IQR 123.6%; *p* < 0.01; dabigatran: 148; IQR 87.5 vs. 192.0; IQR 112.0%; *p* < 0.01) (Figure 7).

**Figure 8:**
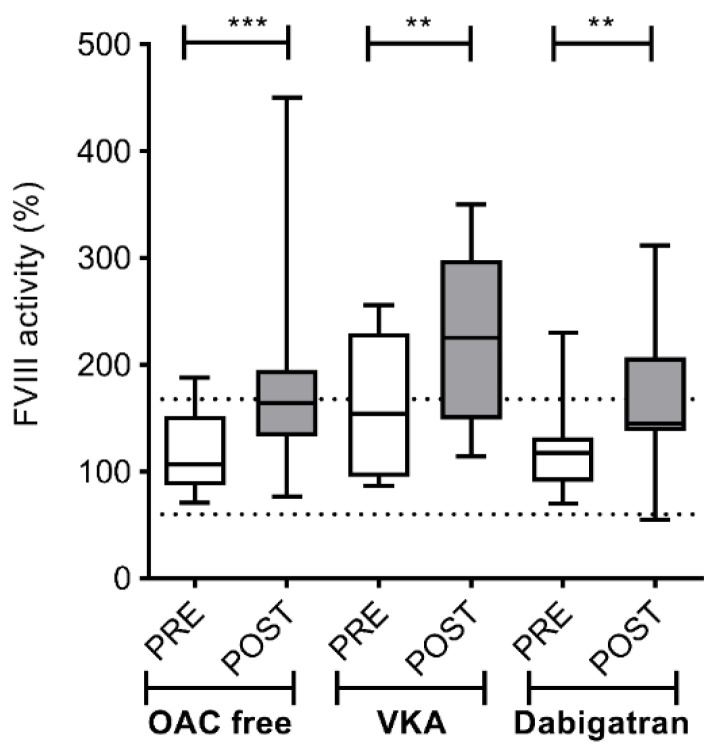
Left atrium FVIII activity levels before and after cryoballoon ablation procedure in AF patients on various pre-procedural anticoagulation strategies. Box and whisker plots indicate median, interquartile range, and total range. Dashed lines indicate lower and upper limit of reference interval (60–168%). PRE (empty bars): pre-ablation, POST (solid bars): post-ablation, OAC: oral anticoagulant, VKA: vitamin K antagonist. ** *p* < 0.01, *** *p* < 0.001.

As expected, FVIII activity showed a similar pattern to what was observed in case of VWF antigen levels, with no difference amongst groups of various pre-procedural anticoagulation strategies (OAC free: 107; IQR 72 vs. 164; IQR 62.25%; *p* < 0.001; VKA: 154; IQR 135 vs. 226; IQR 149%; *p* < 0.01; dabigatran: 110; IQR 32 vs. 144; IQR 88.5%; *p* < 0.01) (Figure 8).

A table summarizing the extent of intracardiac hemostasis activation and endothelial damage related to cryoballoon ablation procedure in the different treatment groups is provided as Table S1 in the Supplementary Materials.

## 4. Discussion

Our results confirm that pre-ablation OAC treatment provides significant inhibition of hemostasis activation triggered by left atrial ablation with the second generation cryoballoon, a technology widely used for transcatheter treatment of AF today. Moreover, our study is the first to provide a complex hemostasis analysis of the left atrium indicating that during cryoballoon catheter ablation, dabigatran provides greater inhibition against intracardiac activation of hemostasis and consequent fibrinolysis as compared to VKAs. We demonstrated that in patients treated with dabigatran, inhibition of local, intra-atrial coagulation activation related to the ablation procedure is markedly suppressed. Indeed, levels of D-dimer, the most commonly used marker to detect a prothrombotic state, were kept below the generally used cut-off of hypercoagulation (0.5 mg/L) in both pre- and post-ablation intracardiac blood samples by dabigatran only, but not in patients treated with VKA or those who received no anticoagulation before the procedure. Similar results were obtained when evaluating a complex set of heparin-insensitive markers of hemostasis/fibrinolysis activation. The level of fibrin monomers, a highly sensitive marker of prothrombotic hemostasis balance was strikingly low in dabigatran-treated patients post-ablation. Although less sensitive markers indicating consumption of hemostasis or fibrinolysis factors do not necessarily show the subtle difference between different anticoagulant regimens, those still demonstrated a considerable activation of hemostasis in patients without pre-procedural anticoagulation, despite receiving heparin during the ablation procedure. Overall, the comprehensive evaluation of our results suggests that dabigatran has the strongest potential to prevent the hypercoagulable state related to a left atrial ablation procedure, while patients undergoing AF ablation with effective VKA anticoagulation might still be exposed to significant hemostasis activation. Moreover, marked coagulation activation was observed in patients receiving no pre-ablation OAC therapy, supporting previous clinical observations on the increased hypercoagulable state carrying a potential risk of thromboembolic events in these patients.

CB ablation induced significant endothelial activation as indicated by increased VWF antigen levels and FVIII activity in the post-ablation blood samples, regardless of pre-ablation anticoagulation treatment. These results suggest that the ablation technology and the area covered by the ablation might be the dominant driver of endothelial damage and antithrombotic therapy per se has a limited potential to mitigate endothelial activation. These data also imply that procedural circumstances were similar in all three groups. Of note, in an earlier work, our group compared three ablation technologies with different biophysical characteristics (focal radiofrequency, multipolar phased radiofrequency, and cryoballoon ablation) using uninterrupted VKA treatment in all patients, and demonstrated no significant differences in the level of endothelial activation [22]. Importantly, in line with the current study, we measured post-ablation D-dimer levels above the 0.5 mg/L cutoff value, in the range usually observed during thromboembolic events. Taken together, this data set could imply that pre-ablation antithrombotic therapy with VKA, which still represents standard practice in many ablation centers, may not provide sufficient control of hemostasis activation in the context of AF ablation.

Antithrombotic therapy with NOACs has evolved as an alternative to VKA in AF patients based on the results of large-scale phase three clinical trials [25,26,27,28]. The extrapolation of these results suggests that the use of NOACs might be advantageous in the periablation setting as well. Administration of these compounds for periprocedural thrombosis prevention in patients undergoing AF ablation is intriguing as many AF patients are already treated with one of these drugs at the time of referral to ablation, thereby making uninterrupted administration of the same drug a simple and pragmatic choice. Related to our research, it has been shown that dabigatran administered to AF patients at a dose of 150 mg was associated with lower rates of systemic stroke and embolism as compared to warfarin during a median of two years follow-up period [25]. Despite the different study design, this finding is in line with our results, demonstrating that dabigatran provided more efficient inhibition against coagulation activation related to endothelial damage as compared to VKAs.

Results of an earlier report on pre-ablation use of dabigatran based on data from a prospective multicenter registry [16] were less encouraging, as dabigatran increased both thromboembolic and bleeding complications as compared with propensity-matched patients who underwent the ablation on uninterrupted warfarin therapy. Importantly, dabigatran dose was withheld on the morning of the procedure in these patients. On the contrary, significantly lower incidence of major bleeding was reported with dabigatran as compared with warfarin (1.6 vs. 6.9%) in the RE-CIRCUIT trial [17], which was a multicenter randomized comparison of uninterrupted warfarin and uninterrupted dabigatran regimen (with the scheduled dose taken on the morning of the procedure) in 704 patients undergoing focal radiofrequency ablation for paroxysmal/persistent AF. In another randomized study (18), the use of interrupted (1–2 doses skipped before the ablation) dabigatran was also associated with significantly less major bleeding as compared with uninterrupted warfarin therapy (1.4% vs. 5.0%). Warfarin was also compared with the FXa inhibitor rivaroxaban [12], edoxaban [15] and apixaban [13,14] in prospective randomized trials and low event rates with no significant differences were reported. FXa inhibitors were administered in an uninterrupted manner in all of these studies with the daily dose of rivaroxaban and edoxaban administered within 18 h before ablation and apixaban taken in the morning of the procedure.

The results of these randomized trials suggest that NOACs administered before AF ablation are safe with bleeding events, comparable to or even lower than with warfarin therapy. This is relevant, as serious bleeding complications may occur during these interventions and a specific antidote for immediate reversal of anticoagulation is currently available for dabigatran only. Of note, the rate of manifest thromboembolic complications related to AF ablation is below 1% in experienced centers [8]; therefore, none of these trials were statistically powered to detect a significant difference in the efficacy endpoint. In our study of a limited number of patients, no bleeding complication was encountered. In the setting of our research, we did not observe any hemostasis or fibrinolytic markers, suggesting a potential risk of bleeding complication in any of the groups (e.g., fibrinogen, α_2_-PI activity, VWF antigen levels, and FVIII activity were all within or above reference range); however, it must be noted that an objective laboratory evaluation of bleeding risk was beyond the scope of the study, as all patients received pre-ablation heparin, prohibiting a comprehensive laboratory testing.

In our investigation, special care was taken to perform all procedures under peak dabigatran concentration based on the pharmacokinetics of the drug. This was ensured by providing a 150 mg tablet to all patients by a nurse 2 h before the start of the procedure and confirmed by drug levels in the therapeutic range as evidenced by measurements taken from blood samples immediately before the first ablation. Our data provide additional laboratory evidence and might complement the results of large-scale randomized trials in AF patients undergoing ablation procedures by supporting data on efficient inhibition of coagulation activation by dabigatran when drug levels are in the therapeutic range. Similar studies on hemostasis parameters with FXa inhibitors as well as with other ablation technologies might also be relevant to supplement available clinical data.

## 5. Conclusions

Uninterrupted administration of dabigatran 150 mg BID before pulmonary vein isolation with the second generation cryoballoon provides efficient inhibition of coagulation activation before and after ablation, as evidenced by a complex panel of hemostasis activation markers measured in left atrial blood samples.

Limitations: This was a non-randomized, observational study. Patients were assigned to any of the three pre-ablation anticoagulation treatment schemes according to the medication they received at the time the ablation procedure was scheduled, which is a common practice in many centers. Importantly, no difference in baseline clinical and relevant procedural parameters was detected between treatment groups. Moreover, after controlling for covariates associated with selection in the statistical model, statistical differences remained the same. Patients in this research had low CHA_2_DS_2_-VASC-scores representing a typical patient cohort undergoing ablation for paroxysmal/persistent AF. The size of the study population was relatively small, but unprecedented, including patients who had AF ablation with such a rigorous pre-ablation anticoagulation protocol and blood sampling from the LA. Although the clinical significance of the measured hemostasis parameters is speculative, our results might provide some guidance for clinicians, while statistically robust data from large-scale clinical research are awaited.

## Figures and Tables

**Table 1:** Baseline clinical and procedural parameters.

	OAC Free (*n* = 24)	VKA (*n* = 11)	Dabigatran (*n* = 17)	*p*
Age (years)	51.2 ± 12.6	58.3 ± 10.8	56.3 ± 10.2	0.240
Male, *n* (%)	15 (62.5)	8 (72.7)	11 (64.7)	0.838
BMI (kg/m^2^)	29.03 (26.68–31.49)	29.02 (28.35–31.47)	26.88 (24.89–30.93)	0.558
Hypertension *n* (%)	13 (54.2)	7 (63.6)	10 (58.89)	0.489
Hypercholesterolemia *n* (%)	13 (54.2)	8 (72.7)	7 (63.6)	0.112
Smoking *n* (%)	7 (29.2)	2 (18.2)	4 (23.5)	0.479
Diabetes mellitus *n* (%)	1 (4.2)	2 (18.2)	1 (5.9)	0.386
Left atrium size (mm)	40.54 ± 5.4	42.30 ± 3.30	41.3 ± 3.6	0.597
Left ventricular ejection fraction (%)	57.38 ± 5.9	60.80 ± 8.04	55.93 ± 6.77	0.228
CHA_2_DS_2_-VASC, median (IQR)	1 (0–2)	1 (0–3)	1 (0–2)	0.780
INR before intervention	0.96 ± 0.05	2.33 ± 0.32	1.18 ± 0.09	<0.001
Dabigatran peak (ng/mL), median (range)	-	-	165.6 (70.6–331.9)	-
hsCRP (mg/L), median (IQR)	1.4 (0.5–2.5)	1.0 (0.5–2.5)	1.7 (0.8–4.6)	0.338
Duration of the procedure (min)	70.08 ± 15.7	78.9 ± 33.7	65.5 ± 22.8	0.155

CHA_2_DS_2_-VASC: Congestive heart failure, Hypertension, Age ≥ 75 years, Diabetes mellitus, Stroke/transient ischemic attack/thromboembolism, Vascular disease, Age (65–74 years), Sex Category (female), INR: International Normalized Ratio, hsCRP: high sensitivity C-reactive protein measurement, IQR: interquartile range, OAC: oral anticoagulant, VKA: vitamin K antagonist.

## Supplementary Materials

The following are available online at https://www.mdpi.com/2077-0383/9/9/3050/s1, Table S1: Summary of intracardiac hemostasis activation and endothelial damage related to cryoballoon ablation procedure in patients with atrial fibrillation according to various pre-procedural anticoagulation strategies.
