# Supplementary material for: Uninterrupted Dabigatran Administration Provides Greater Inhibition against Intracardiac Activation of Hemostasis as Compared to Vitamin K Antagonists during Cryoballoon Catheter Ablation of Atrial Fibrillation
Source: J Clin Med. 2020 Sep 22;9(9):3050. doi: 10.3390/jcm9093050 (PMC7563747; doi:10.3390/jcm9093050)
Supplement: Supplementary file 1 [file jcm-09-03050-s001.pdf]

Journal of
Clinical Medicine

Supplementary Table 1. Summary of intracardiac hemostasis activation and endothelial damage related to cryoballoon ablation procedure in patients with atrial fibrillation according to various pre-procedural anticoagulation strategies.

Markers of intracardiac hemostasis activation	OAC free	VKA	Dabigatran
D-dimer:			
Pre-ablation median value above the upper limit of reference	no	no	no
Post-ablation median value above the upper limit of reference	yes	yes	no
Significant difference between pre-ablation vs. post-ablation levels	yes	yes	yes
PAP-complex:			
Significant difference between pre-ablation vs. post-ablation levels	yes	yes	no
α2-plasmin inhibitor activity:			
Significant difference between pre-ablation vs. post-ablation levels	yes	no	no
Fibrinogen:			
Significant difference between pre-ablation vs. post-ablation levels	yes	no	no
Fibrin-monomer:			
Pre-ablation median value above the upper limit of reference	yes	yes	yes
Post-ablation median value above the upper limit of reference	yes	yes	no
Significant difference between pre-ablation vs. post-ablation levels	yes	yes	yes
**Markers of endothelial damage**	**OAC free**	**VKA**	**Dabigatran**
VWF antigen:			
Pre-ablation median value above the upper limit of reference	no	no	no
Post-ablation median value above the upper limit of reference	yes	yes	yes
Significant difference between pre-ablation vs. post-ablation levels	yes	yes	yes
FVIII activity:			
Pre-ablation median value above the upper limit of reference	no	no	no
Post-ablation median value above the upper limit of reference	no	yes	no
Significant difference between pre-ablation vs. post-ablation levels	yes	yes	yes

OAC: oral anticoagulant, FVIII: factor VIII, PAP complex: plasmin-antiplasmin complex, VKA: vitamin K antagonist, VWF: von Willebrand factor.
